# Identification of differential immune cells and related diagnostic genes in patients with diabetic retinopathy

**DOI:** 10.1097/MD.0000000000035331

**Published:** 2023-09-29

**Authors:** LinHui Yuan, LiJun Zhang, Xin Liu, Sheng Li, JiXin Zou

**Affiliations:** a Department of Ophthalmology, the Third People’s Hospital Affiliated to Dalian Medical University, Dalian, Liaoning, China; b Dalian Medical University, Dalian, Liaoning, China.

**Keywords:** biological markers, diabetic retinopathy, differentially expressed genes, enrichment analysis, immunity

## Abstract

**Background:**

Diabetic retinopathy (DR) is a frequent microvascular abnormality associated with diabetes mellitus. The loss of retinal immunity is an important underlying mechanism of the DR pathogenesis, including the change in retinal immunosuppressive characteristics and the blood-retinal barrier disturbances. Therefore, this investigation screens immune-associated biomarkers in the retina of DR patients.

**Methods:**

In this investigation, the differential expression genes (DEGs) were acquired from Gene Expression Omnibus data GSE102485. The relative expression of 22 immune cell types in each sample was calculated by CIBERSORT analysis based on gene expression profile. The core module closely associated with immune infiltration was also screened by weighted gene co-expression network analysis (WGCNA). The overlapping DEGs and module genes were the differentially expressed immune-related genes (DEIRGs). With the help of the genes/proteins (STRING) database and MCODE plug-in, the protein-protein interaction (PPI) network hub genes were screened. Furthermore, the miRNA—hub genes and transcription factor (TF)—hub gene regulatory network were used to explain the possible signal pathways in DR. The hub genes verification was carried out by Polymerase Chain Reaction. Lastly, select CSF1R and its related pathway factor p-ERK1/2 to verify their expression in RF/6A under normal and high glucose environments.

**Results:**

A total of 3583 principle DEGs, that enriched immune-related GO terms and infection-related pathways were identified. CIBERSORT analysis showed that naive B cells, M2 macrophages, eosinophils, and neutrophil infiltration were significantly different. After intersecting 3583 DEGs, 168 DEIRGs and 181 module genes were identified. Furthermore, 15 hub genes, TYROBP, FCGR3A, CD163, FCGR2A, PTPRC, TLR2, CD14, VSIG4, HCK, CSF1R, LILRB2, ITGAM, CTSS, CD86, and LY86, were identified via PPI network. The identified hub genes were up-regulated in DR and showed a high DR diagnostic value. Regulatory networks of the miRNA- and TF-hub genes can help understand the etiology of disease at the genetic level and optimize treatment strategy. CD14, VSIG4, HCK, and CSF1R were verified to be highly expressed in the vitreous of patients with DR. n RF/6A, CSF1R, and p-ERK1/2 were significantly overexpressed under high glucose conditions, with a statistically significant difference.

**Conclusion:**

This investigation identified 15 genes (TYROBP, FCGR3A, CD163, FCGR2A, PTPRC, TLR2, CD14, VSIG4, HCK, CSF1R, LILRB2, ITGAM, CTSS, CD86, and LY86) as hub DR genes, which may serve as a new potential point for the diagnosis and treatment of DR. CSF1R/p-ERK1/2 signaling may promotes the development of retinal neovascularization.

## 1. Introduction

Diabetic retinopathy (DR) is a retinal vascular disease. In China, it is most frequently found in individuals of working age. DR is a progressive and specific complication of diabetes. Irreversible retinal damage caused by high glucose is characterized by microaneurysms, capillary occlusion, fibrosis, and neovascular proliferation.^[1]^ Its treatment is complicated and refractory, and the cycle is long. It has become one of the most intractable ophthalmology diseases worldwide in recent years. Therefore, the determination of genetic markers and therapeutic targets to provide new methods for the treatment and intervention of DR is very important.

The retina is an immune-privileged tissue. The blood-retina-barrier (BRB) and immune suppressive microenvironment protect the retina against external and internal intrusion and attack.^[2]^ Together the immune-competent cells, mediators, and the complement system participates in the immune dysregulation of DR, causing the dysregulation of retinal homeostasis.^[3]^ In DR, because of extensive BRB damage, the retinal immunity is disrupted, the immune tolerance is reduced, the immune deviation is lost, and a large number of circulating immune cells infiltrate the retina and activate innate/acquired immunity, leading to progressive degeneration of retinal vessels.^[4]^ Microglia have a phagocytic function and are an important part of the innate immune system. The retina and optic nerve serve as the epitaxy of the CNS. Microglia play a crucial part in the immunological pathogenesis of eye diseases, for instance, they participate in the pathogenesis of DR by phagocytosing RGC, secreting cytotoxic substances, and disrupting the structure and function of BRB.^[5]^ Studies have shown that retinal vascular damage in DR is closely associated with complement system activation.^[6]^ The high glucose environment markedly stimulates the expression of local and systemic chemokines and inflammatory factors, and the persistent mild inflammation that results from it is thought to promote the associated damage to the vascular and immune system in DR, inducing BRB destruction, further causing macular edema and retinal neovascularization.^[7]^

The aforementioned literature suggests that immunity is a key factor in DR. Therefore, this investigation aims to find immune-related markers in DR.

## 2. Materials and Methods

### 2.1. Determination of differential expression genes (DEGs)

The gene expression profile GSE102485 was acquired from the Gene Expression Omnibus database. Twenty-one transcriptome profiles of neovascular proliferative membrane from 19 DR and 3 normal samples were selected. DEGs were obtained by R package “DESeq2.”^[8]^ The adj.p.val < 0.05 and |log_2_FC| > 1 were set as the cutoff criterion for DEGs.

### 2.2. Biological function analyses

Gene ontology (GO) and Kyoto Encyclopedia of Genes and Genomes (KEGG) pathway enrichment analyses were carried out with the help of the R package “Org.Hs.e.g.db.”^[9]^ The GO annotation contained 3 biological aspects: cellular component, biological process, and molecular function.

### 2.3. Analysis of immune cell characteristics

The “CIBERSORT” R package was utilized to determine the proportion of 22 infiltrating immune cell subtypes in the DR and normal cohort and this proportion was predicted by the relative gene expression levels in individual tissue samples based on their gene expression profiles.^[10]^ Differential infiltrating immune cells between DR and normal samples (adj.p.val < 0.05) were identified for further analysis.

### 2.4. Weighted gene co-expression network construction and analysis (WGCNA)

The R package “WGCNA” was applied to identify differential infiltrating immune cells related modules and genes.^[11]^ At first, hierarchical clustering was performed on the sample to detect and eliminate outliers. WGCNA algorithm uses the correlation coefficients’ weighted value to form connections between genes in the network and obey scale-free networks. The pickSoftThreshold function in the “WGCNA” package is used to determine the soft threshold power. The correlation coefficient *R*^2^ > 0.85 as a soft threshold β Standards.

### 2.5. PPI network construction and hub genes identification

The PPI network of the 168 differentially expressed immune-related genes (DEIRGs) was established online by the STRING database, and the isolated network nodes were eliminated.^[12]^ Subsequently, the core PPI network module was screened by the Cytoscape software plugin Molecular Complex Detection (MCODE) with the following default parameters: node score cutoff = 0.2, degree cutoff = 2, k-score = 2, and max. Depth = 100.^[13]^ The genes in the core module were the hub genes.

### 2.6. Verification of hub genes by receiver operating characteristic (ROC) analysis

To verify the diagnostic significance of 15 hub genes, the ROC curve was graphed via the “pROC” package to determine the ability to distinguish DR and normal tissues.^[14]^ A higher AUC value represented a better ability of the hub gene to distinguish whether the sample was diseased or not.

### 2.7. Construction of the regulatory network

To predict the miRNAs and transcription factors (TFs) that target hub genes, an online visual assessment tool; the miRNet database was utilized.^[15]^ Then based on the miRNAs, TFs, and hub genes, the miRNA- and TF-hub gene regulatory networks were constructed using Cytoscape software.

### 2.8. Sampling

Five hub genes were selected for verification: CD168, CD14, CSF1R, HCK, and VSIG4. This research scheme was authorized by the Ethics Committee of Dalian Third People Hospital (Approval No.: 2022-096-001), and all patients signed the informed consent form. This is a prospective nonrandomized controlled study. Twenty patients (20 eyes) with proliferative DR and twenty patients (20 eyes) with an idiopathic macular hole or idiopathic macular epiretinal membrane who underwent vitrectomy in our hospital from September 1 to December 31, 2022, were selected, regardless of gender. Exclusion parameters included: Patients with malignant tumors. Patients with other eye diseases, such as eye trauma history. Pregnant patients. Patients who did not reach 18 years of age. Patients who showed signs of systemic and ocular infection, fever, or other stress states within 24 hours. Patients with hematologic diseases. Lastly, routine blood tests, electrocardiograms, and other related cardiac function tests were conducted for all patients, and those who were unable to receive surgical treatment because of other systemic diseases or whose surgical treatment is limited were also excluded. A vitrectomy was performed by the same surgeon. Briefly, after 3 standard scleral incisions, a 1 mL needle tube behind the Vitreous cutting handle was connected, then with the help of an assistant, after the perfusion closed, the vitreous body was incised, simultaneously the assistant slowly absorbs 0.5 mL of the vitreous body and saved it in the sterile EP tube. Then the surgeon completed the operation.

### 2.9. Quantitative real-time reverse transcriptional polymerase chain reaction (q-RT-PCR)

According to the human gene coding sequence in the GenBank database, Primer 5.0 software was utilized for designing specific forward and reverse primers and the primer sequences were synthesized by Beijing Tianyihuiyuan Biotechnology Co., Ltd. The gene (with Actin as the internal reference gene) and primer information are given in Table 1. Actin was set as an endogenous variable to correct individual differences. 2-ΔΔCt was used as the basis for statistical evaluation, and via the GraphPad Prism6 the expression differences among different tissues were analyzed.

**Table 1:** Gene and primer sequences for PCR.

Gene	Primer sequence
CSF1R	F: CAACGGCTCTGGCACCC R: GCACCGTCACCTTGTGGAA
VSIG4	F: ATGAGCCAACTTCCCAGAATCT R: GGCAAGGACTGACTAGGCAATT
HCK	F: GCCATTCACCACGAAGACC R: GCGGGCGACATAGTTGCTT
CD14	F: GGCGAACGCGGACTGAT R: AGCGAACGACAGATTGAGGG

### 2.10. Expression of CSF1R and p-ERK1/2 in RF/6A under normal and high glucose environments

The rhesus monkey chorioretinal endothelial cells (RF/6A, Saibaikang Biotechnology Co., Ltd.) were routinely cultured. After the fusion degree of the cells reached the requirements, the high sugar culture medium containing 25 mM glucose was replaced, and the culture continued for 48 hours. The high glucose culture group was HG group, and the normal culture control group was Control group. Immunocytochemistry (ICC) was used to detect the expression of CSF1R protein. Real time PCR was used to detect the expression of CSF1R and p-ERK1/2 mRNA. Western blot was used to detect the expression of CSF1R and p-ERK1/2 protein.

#### 2.10.1. ICC.

Use 0.1% Triton × Incubate at room temperature for 20 minutes at 100 °C, and incubate at room temperature with 3% H2O2 for 15 minutes to eliminate the activity of endogenous peroxidase. Add 1% BSA, incubate at room temperature for 15 minutes, pour out the serum, dilute the first antibody with PBS at 1:100, drip until the cells are completely covered, and spend the night at 4 °C. HRP labeled goat anti rabbit was diluted 500 times with PBS, added dropwise to completely cover the cells, and incubated at 37 °C for 60 minutes. Remove the secondary antibody from the 12-hole plate. After that, DAB coloration and hematoxylin re staining were performed, and the staining effect was observed under a microscope. Take photos under a mirror.

#### 2.10.2. Real time PCR.

The method is the same as that in 2.9, The gene (with Actin as the internal reference gene) and primer information are given in Table 2.

**Table 2:** Gene and primer sequences for PCR.

Gene	Primer sequence
CSF1R F	CGTGCTGTTGACCAATG
CSF1R R	TCGCTCTGAACCGTGTA
β-actin F	GGCTCCGGCATGTGCAA
β-actin R	CGATGGGGTACTTCAGGGTG

#### 2.10.3. Western blot.

Perform a BCA reaction on the extracted protein, set the reading wavelength to 570 nm for reading, and record the data. Draw a standard curve based on the standard protein concentration and corresponding absorbance value, calculate the sample protein concentration through a regression equation, and multiply by the dilution factor to obtain the protein concentration of the sample. After performing an SDS-PAGE reaction, transfer printing is performed. After sealing with a skimmed milk powder solution, the first and second antibodies are incubated, and ECL substrate luminescence is performed. They are transferred to a cassette and exposed in a dark room. The film is scanned, and the optical density value of the target strip is analyzed with the gel image processing system (Gel-Pro-Analyzer software).

### 2.11. Statistical analysis

In this experiment, RT-PCR results were analyzed using Actin as the internal parameter to correct for individual differences, and statistical analysis was conducted based on 2-ΔΔ CT. Western Blot results were analyzed using image J software to analyze the band gray value. The target band gray value was compared with the internal parameter band gray value, and the relative expression of the target protein was calculated. Cell immunohistochemistry results were calculated using image J software to calculate the average optical density of cells, and the average optical density of HG group and Control group was compared, Calculate the relative expression of the target protein. All results were analyzed for expression differences between different tissues using GraphPad Prism9, and all data were expressed as mean ± standard deviation (SD). Single factor analysis of variance was used for the difference test, and the minimum significant difference t-test was used to compare the data between each 2 groups. *P* < .05 was considered statistically significant.

## 3. Results

### 3.1. Identification of DEGs in DR

**Figure 1.**
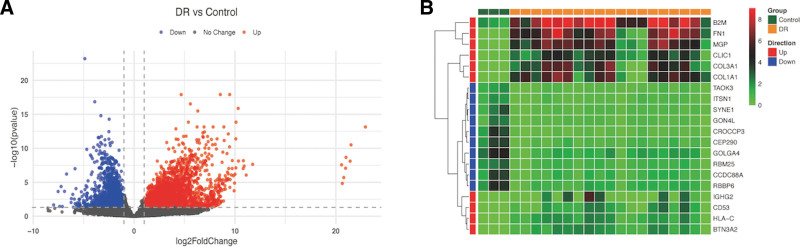
(A) Volcano plot for the differential expression analysis. DEGs were selected with p.val < 0.05 and |log2FC| > 1 among the the gene expression profile of GSE102485. (B) Heatmap for DEGs. Heat map of the 10 most significantly up-regulated genes and the 10 most significantly down-regulated genes (sort according to the corrected *P* value). DEGs = differential expression genes.

Using adj.p.val < 0.05 and |log_2_FC| > 1 as a cutoff parameter, 3583 DEGs were determined from the GSE102485 dataset. There were 1185 up-regulated and 2398 down-regulated genes in DR tissues in contrast to normal ones (Fig. 1A). The expression heatmap of the top 10 up-regulated and down-regulated DEGs (sorted according to the adj.p.val) is presented in Figure 1B.

### 3.2. GO term and KEGG pathway enrichment analyses of DEGs

**Figure 2.**
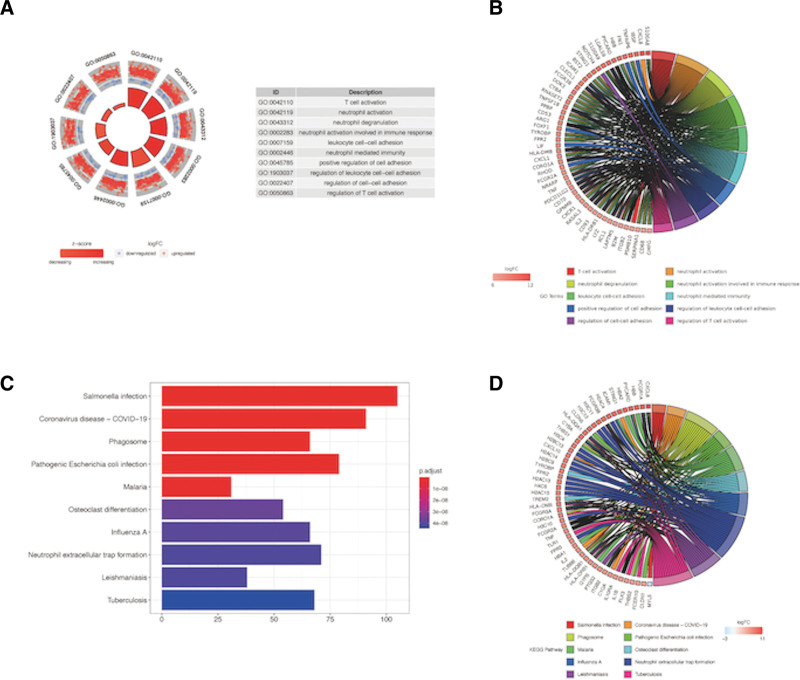
(A) Circle plot for GO enrichment of DEGs. (B) Chord plot for GO enrichment of DEGs. (C) Bar plot for KEGG enrichment of DEGs. (D) Chord plot for KEGG enrichment of DEGs. DEGs = differential expression genes, GO = Gene ontology.

The R package “Org.Hs.e.g.db” revealed that the DEGs were specifically enriched in immune-associated GO terms such as “T cell activation,” “neutrophil activation,” “neutrophil degranulation,” “neutrophil activation involved in immune response” and “leukocyte cell-cell adhesion” (Fig. 2A and B). As to KEGG pathway analysis, DEGs were mostly associated with infection, for example, “Salmonella infection,” “coronavirus disease—COVID-19,” “phagosome,” “pathogenic *Escherichia coli* infection” and “malaria” (Fig. 2C and D).

### 3.3. Infiltrating immune cell analysis

**Figure 3.**
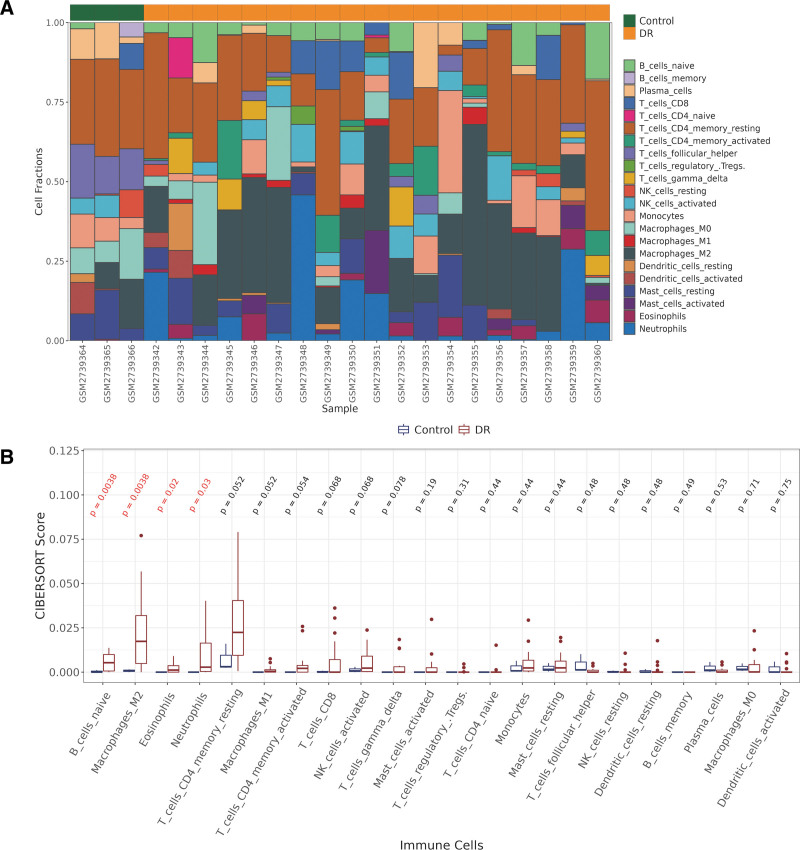
(A) Stack plot for CIBERSORT. (B) Box plot for CIBERSORT. The Wilcoxon test indicated that naive B cells, M2 macrophages, eosinophils, and neutrophil infiltration had notably higher enrichment in DR tissues (*P* < .05). DR = diabetic retinopathy.

The immune cell composition in each sample was evaluated by CIBERSORT (Fig. 3A). The variability of immune cell infiltrations in the DR and normal cohort is presented in Figure 3B. The Wilcoxon test indicated that naive B cells, M2 macrophages, eosinophils, and neutrophil infiltration had notably higher enrichment in DR tissues.

### 3.4. WGCNA and identification of the key module

**Figure 4.**
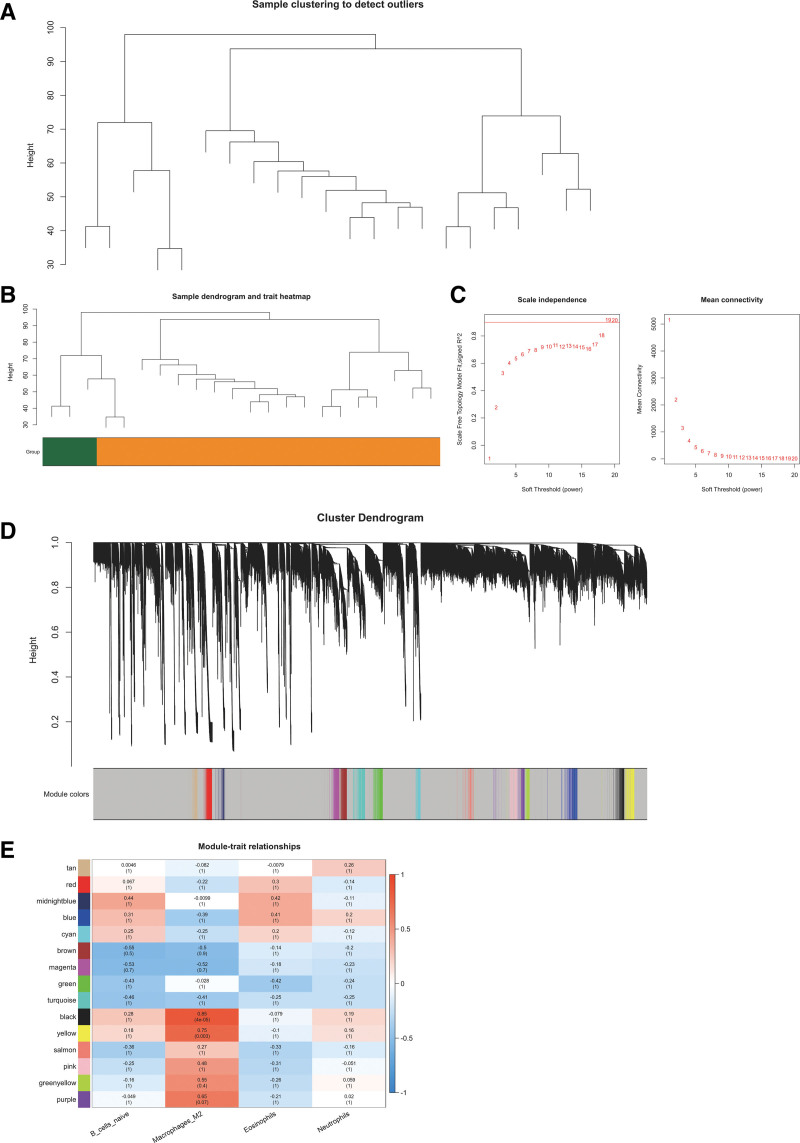
(A) Sample clustering to detect outliers. (B) Sample clustering. (C) Soft thr eshold selection. Calculation chart of adjacency matrix weighting parameters (power). The X-axis represents weighting parameters (power). The Y-axis represents quadratic of correlation index from log (k) and log (P(K)). (D) WGCNA modules. System clustering tree was built based on GSE102485 dataset. Ten kinds of color present 15 modules. (E) Correlation between coexpr ession modules and CIBERSORT scores. Pearson correlation coefficient for each correlation and *P* value in are shown in each cell. WGCNA = weighted gene co-expression network analysis.

Initially, based on the Euclidean distance of the expression the hierarchical clustering was performed, and the results showed no outliers (Fig. 4A and B). Under the soft-thresholding power of 19, 15 modules were identified (Fig. 4C and D). After module-infiltrated immune cells analysis, it was observed that the highest correlation was between the black module and M2 macrophages (*P* = 4 × 10^−5^, Cor = 0.85, Fig. 4E). Consequently, 181 black module genes were determined as immune-related genes for further studies.

### 3.5. DEIRGs screening and enrichment analysis

**Figure 5.**
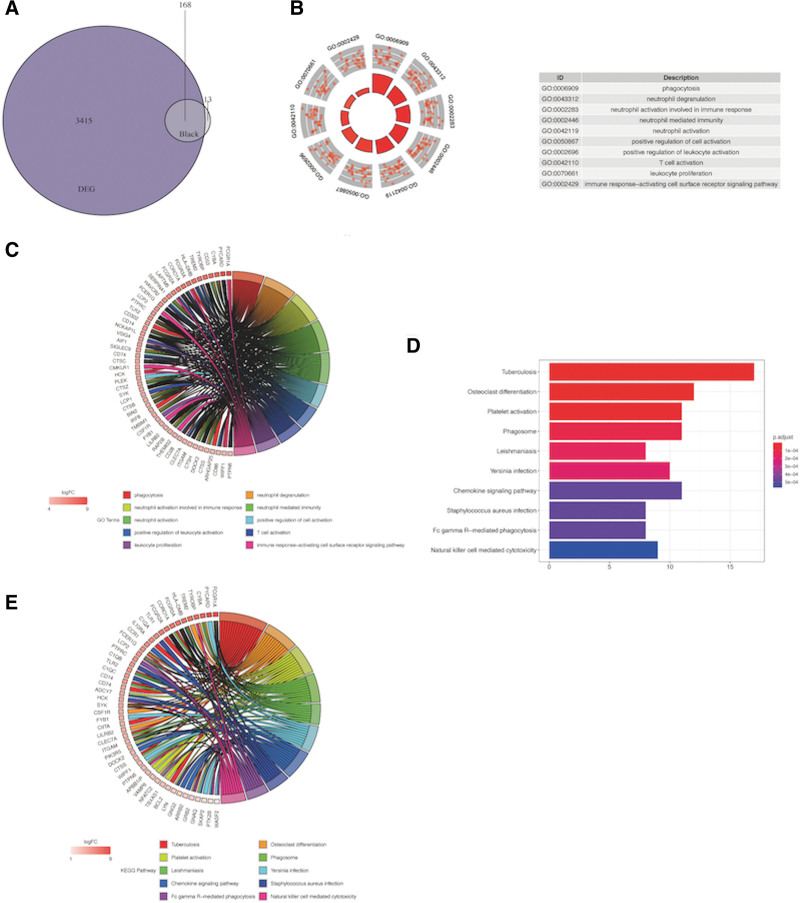
(A) Intersection between genes in module black and DEGs. (B) Circle plot for GO enrichment of 168 genes. (C) Chord plot for GO enrichment of 168 genes. (D) Bar plot for KEGG enrichment of 168 genes. (E) Chord plot for KEGG enrichment of 168 genes. DEGs = differential expression genes, GO = Gene ontology.

The selected DEGs and module genes were intersected, and a total of 168 overlapping genes were screened out as differential immune-related characteristic genes for DR (Fig. 5A). For the GO enrichment analysis, the important terms associated with DEIRGs were “phagocytosis,” “neutrophil degranulation,” “neutrophil activation involved in immune response,” “neutrophil-mediated immunity” and “neutrophil activation” (Fig. 5B and C). And for KEGG enrichment analysis, the significant DEIRGs pathways included “phagosome,” “yersinia infection,” “chemokine signaling pathway,” “*Staphylococcus aureus* infection” and “natural killer cell-mediated cytotoxicity” (Fig. 5D and E).

### 3.6. Determination of hub genes by PPI network

**Figure 6.**
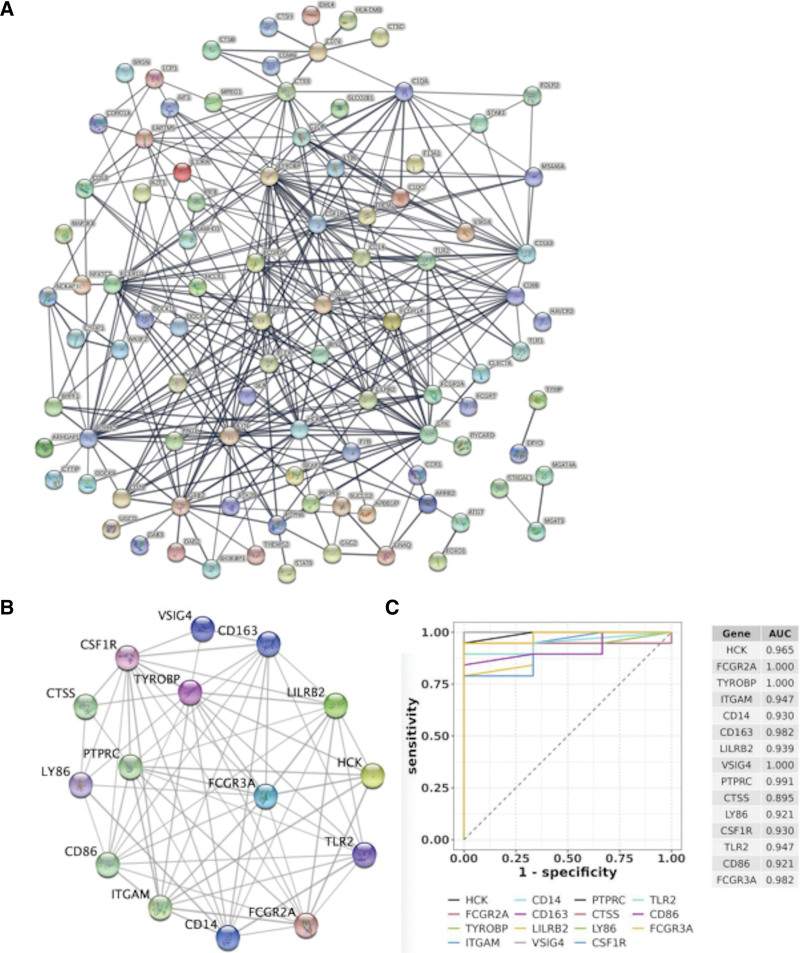
(A) PPI network. The PPI network covers a total of 15 DEGs. (B) subnetwork of hub genes. (C) Expression of hub genes. DEGs = differential expression genes. PPI = protein-protein interaction.

The PPI network of the 168 DEIRGs of DR was established based on the data acquired from the STRING database (Fig. 6A). Then, with the help of the MCODE plugin, the core module was identified from the PPI network. The following 15 genes were extracted as hub genes, TYROBP, FCGR3A, CD163, FCGR2A, PTPRC, TLR2, CD14, VSIG4, HCK, CSF1R, LILRB2, ITGAM, CTSS, CD86, and LY86 (Fig. 6B). And as illustrated in Figure 6C, the 15 hub genes were all up-regulated genes in the DR group.

### 3.7. Verification of hub genes in DR

**Figure 7.**
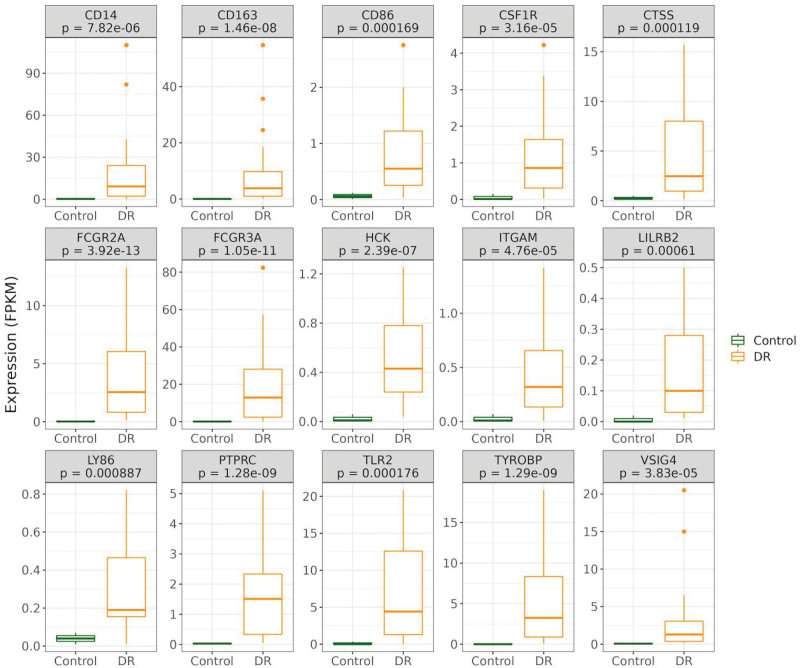
ROC of hub genes on determining DR patients from normal control. DR = diabetic retinopathy. ROC = receiver operating characteristic.

To evaluate the diagnostic ability of the 15-hub gene for DR, ROC curve analysis was performed. AUC values for 15 genes were all >0.85 (Fig. 7). The results demonstrated that the hub genes accurately distinguished DR and the control groups, making these genes valuable parameters for the diagnosis of DR.

### 3.8. Construction of miRNA-hub gene and TF-hub gene regulatory networks

**Figure 8.**
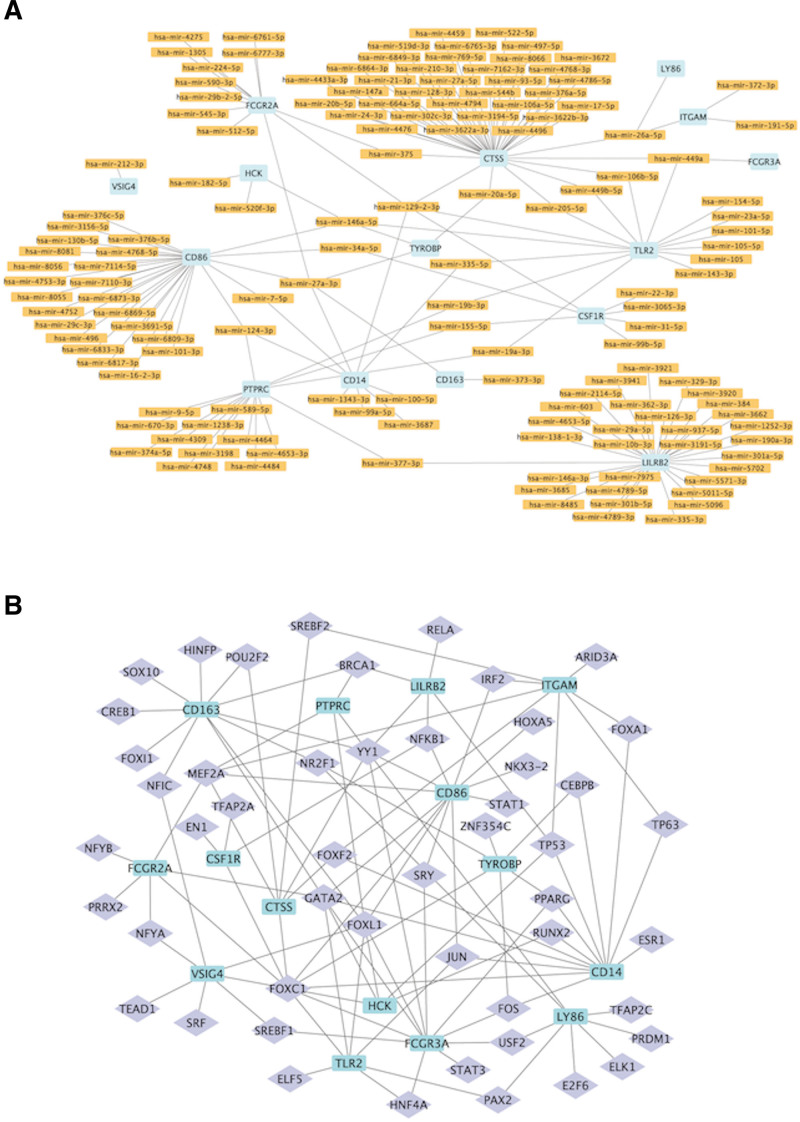
(A) MiRNA regulation network of hub genes. (B) Transcription factor regulation network of hub genes.

The predicted targeting miRNAs on 15 hub genes indicated the identification of 145 miRNAs after the removal of repeating miRNAs. Then, Cytoscape was utilized to describe their relationships in the network. CTSS, LILRB2, and CD86 had a targeted relationship with 42, 32, and 27 miRNAs, respectively (Fig. 8A).

Fifty TFs of hub genes were obtained, then, their regulatory pathway was connected in a network, to reveal the possible regulatory mechanism. The top 4 hub genes were CD14, CD86, CD163, and FCGR3A, which were regulated by 12, 12, 11, and 11 TFs, respectively (Fig. 8B).

### 3.9. RT-PCR validation of the hub genes

**Figure 9.**
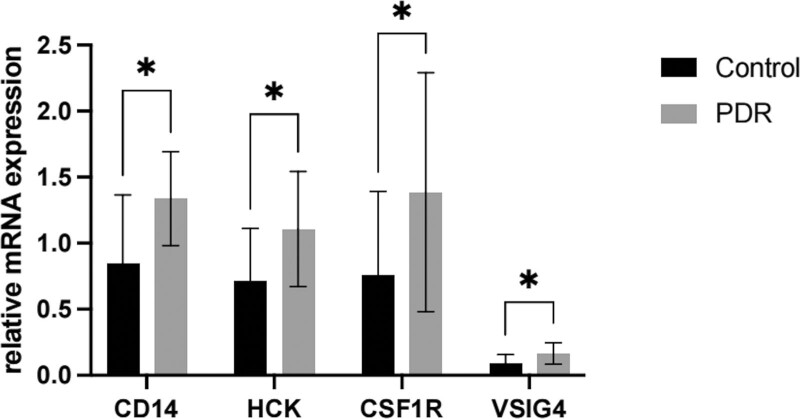
Relative mRNA expression.

The data revealed that CD14, CSF1R, HCK, and VSIG4 had high relative expressed in the DR group, these results are consistent with the previous investigations. Moreover, the relative CD168 expression levels were lower in the DR group, and all the results had significant differences (Fig. 9).

### 3.10. Results of Cellular Immunohistochemical

**Figure 10.**
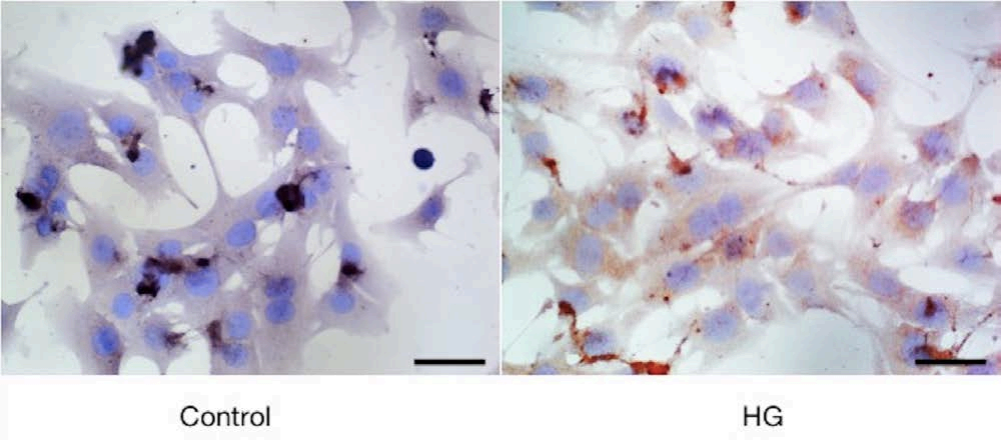
Immunohistochemical results of CSF1R protein in both groups of cells (200×).

**Figure 11.**
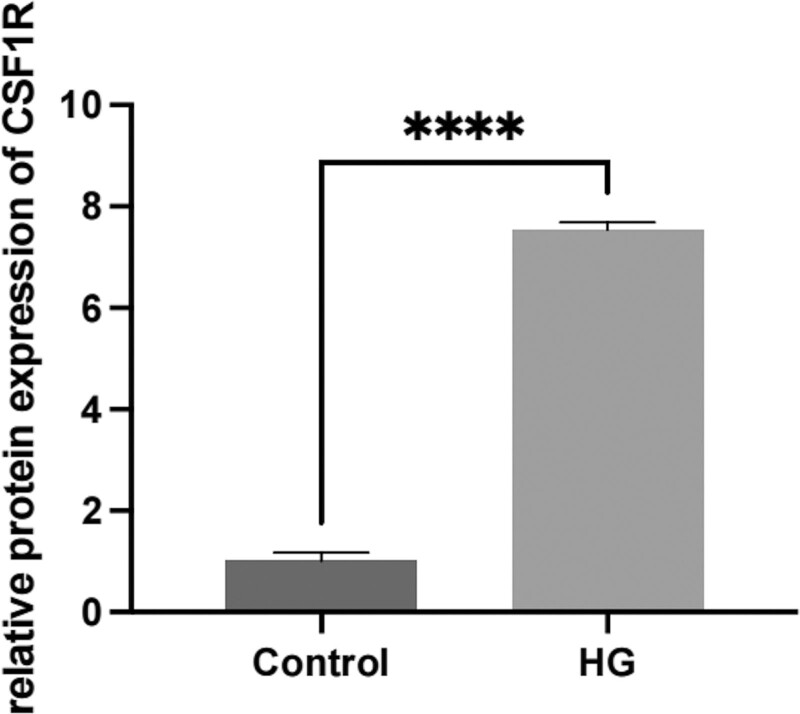
Relative expression level of CSF1R protein in Control group and HG group, ****P < .001.

The results of cell immunohistochemistry showed that blue purple was the nucleus, and brown yellow was the positive expression. The more brown yellow, the more positive expression. The results of immunohistochemistry for CSF1R protein were shown in Figure 10, and the results of relative expression were shown in Figure 11. The relative expression of CSF1R protein in the high glucose treatment group was higher than that in the control group, and the difference was statistically significant (*P* < .05).

### 3.11. Results of relative expression of CSF1R mRNA

**Figure 12.**
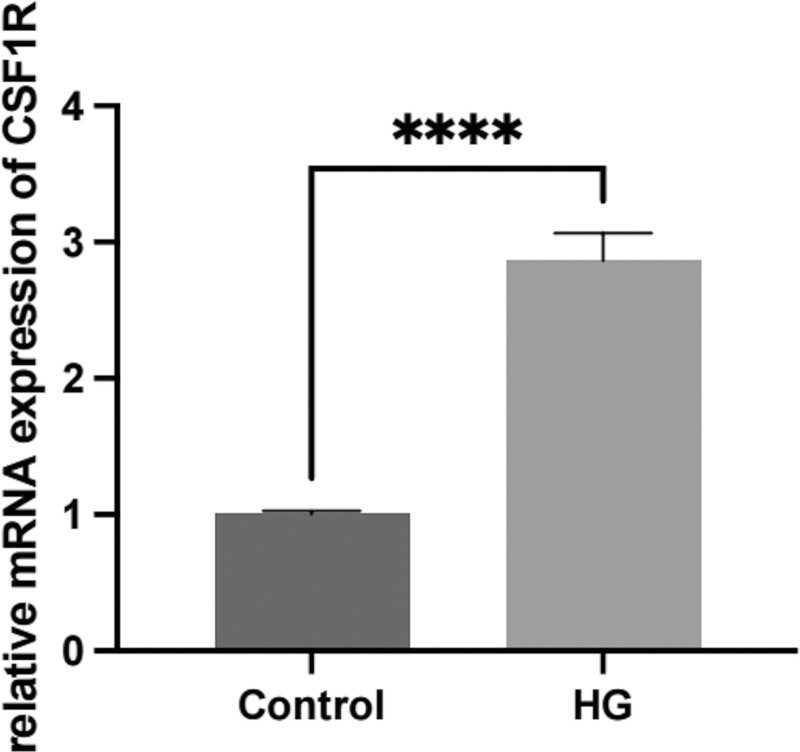
Relative expression of CSF1R mRNA in the 2 groups, ****P < .001.

The relative expression level of CSF1R mRNA in the high glucose group compared to the control group is shown in Figure 12. Compared with the control group, the relative expression of CSF1R mRNA was 2.87 ± 0.20 for the high glucose treatment group and 1.00 ± 0.02 for the control group. The difference in expression between the 2 groups was statistically significant (*P* < .05).

### 3.12. Results of relative expression of CSF1R and p-ERK1/2 proteins

**Figure 13.**
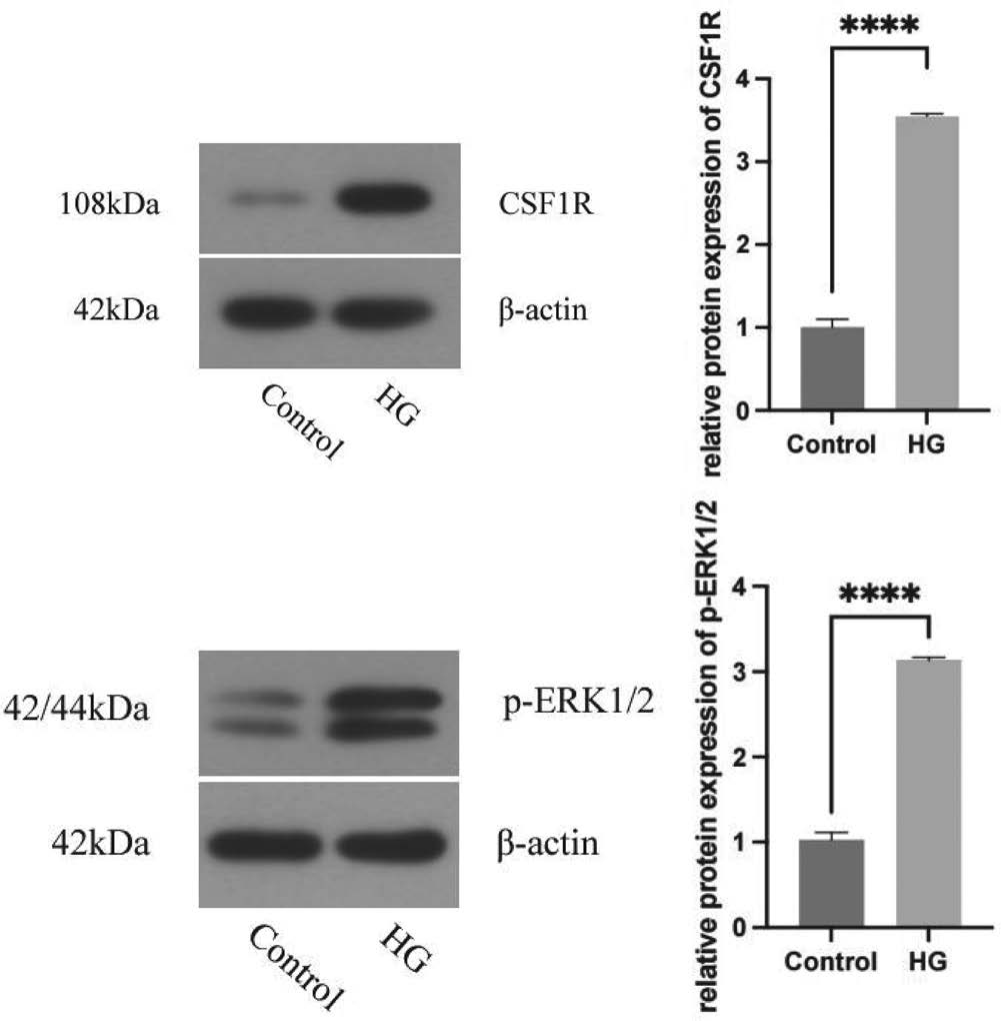
The relative protein expression levels of CSF1R and p-ERK1/2 in the high glucose treatment group and the control group, ****P < .001.

The protein expressions of CSF1R and p-ERK1/2 are shown in Figure 13. The protein content of CSF1R and p-ERK1/2 in the high glucose treatment group was higher than that in the control group, and the difference was statistically significant (*P* < .05).

## 4. Discussion

Most previous studies believed that the pathological process of DR mainly included pericyte loss, endothelial cell proliferation, basement membrane thickening, lumen stenosis or occlusion, retinal ischemia or hypoxia, angiogenesis, etc.^[16]^ However, recently, some scholars indicated the presence of many kinds of autoantibodies in the serum of DR patients, such as anti-aldolase and anti-pericyte antibodies. The effectiveness of antibiotics, immune suppressants, and corticosteroids in DR treatment proved that immune system disorders and inflammation are important pathophysiological factors of DR.^[17–19]^ Given the important role of immunity in DR, this study identified the immune cells and related diagnostic markers of DR patients.

An obvious contrast in mRNA was observed between DR and control samples. Subsequently, enrichment research on DEG was conducted. GO enrichment analysis found important immune-related biological events such as “T cell activation,” “neutrophil activation,” “neutrophil degranulation,” “neutrophil activation involved in immune response” and “leukocyte cell adhesion.” KEGG enrichment analysis shows that differential genes are enriched in infection-related pathways, such as “Salmonella infection,” “coronavirus disease—COVID-19,” “phagosomes,” “pathogenic *E coli* infection” and “malaria.” The aforementioned biological processes and signal pathways might be a crucial part of the pathogenesis of DR Many gross inflammatory cells and inflammatory factors participate in the above biological processes and signal pathways. Hyperglycemia leads to the damage of vascular endothelial cells and the aggregation and adhesion of leukocytes to the vascular wall, an important pathological process that initiates an inflammatory response. Activated leukocytes can not only release a large number of inflammatory factors but also cause hemodynamic changes by aggravating endothelial cell damage. After adhesion of activated neutrophils with endothelial cells, they form an extra neutrophil trap, which limits and clears the injured endothelial cells, leading to circulatory disorders, no perfusion formation, aggravating tissue ischemia and hypoxia, causing vascular remodeling and neovascularization.^[20]^ Various inflammatory chemokines can recruit concentrated granulocytes, monocytes, and lymphocytes to migrate to the inflammatory site and promote activation. Mechanisms such as IL-6 and IL-8 may be related to the activation of mitogen-activated protein kinase, mitogen-activated protein kinase/extracellular regulated protease, and vascular endothelial growth factor pathway.^[21,22]^

The immune characteristics of DR were also analyzed and it revealed a significant difference in the infiltration of M2 macrophage immune cells between DR and the control groups. Many studies have shown that M2 macrophages may participate in proliferative diabetic retinopathy (PDR) angiogenesis.^[23–25]^ The microglia or macrophages were activated in an oxygen-induced retinopathy model. Inhibiting macrophage polarization to M2 phenotype can inhibit the generation of retinal neovascularization, which may be an effective way to treat retinal neovascular diseases.^[26]^ The activation of NLRP3 in the vitreous of PDR patients leads to the increase of M2 macrophages, which stimulate angiogenesis and fibrosis. NLRP3 is a key regulator of 2 innate immunities, so M2 macrophages can aggravate DR, which may be related to innate immune imbalance.^[27]^ The above results indicated that immune infiltration may play an important function in the neovascularization of DR by promoting the polarization of macrophages to the M2 type and the formation of vascular endothelial cell lumen.

In this study, 168 immune-associated genes significantly involved in DR were found by the intersection of DEG and WGCNA module genes. Function and pathway enrichment analysis showed that they were closely related to immunity and infection.

To further search for key genes affecting DR, 15 hub genes were screened by the PPI network: TYROBP, FCGR3A, CD163, FCGR2A, PTPRC, TLR2, CD14, VSIG4, HCK, CSF1R, LILRB2, ITGAM, CTSS, CD86, and LY86. The diagnostic performance of the hub gene was evaluated by the ROC curve and all showed high diagnostic values. CD163 can promote the occurrence and development of DR by promoting inflammatory reactions.^[25,28,29]^ A study showed that CSF1R in the retina of diabetic rats is upregulated as compared with normal rats. Furthermore, the levels of M-CSF in vitreous samples of PDR patients are upregulated as proved by ELISA.^[30]^ CD14 not only aggravates pathological changes by promoting inflammatory reactions in the late stage of DR^[31]^ but also is closely related to macular edema in DR. Compared with patients with focal edema, DME patients with diffuse edema have higher levels of CD14 in the retina.^[32]^ The mechanism of other hub genes in eye diseases has not been reported yet, and their role in DR needs further research.

Additionally, the regulation mechanism of the hub gene was also studied. The identified 15 hub genes are regulated by 145 miRNAs and 50 TFs. The TFs-genes network showed that NF-kB, YY1, NR2F1, MEF2A, and SRY might be a crucial part of DR pathogenesis. HMGB1 may be involved in the pathogenesis of DR as a TF through the NF-kB pathway.^[33]^ TLR4/MyD88/NF-κB p65 pathway participates in the process of DR by regulating the polarization of microglia.^[34]^ MiR-195 increases EMT and cell permeability mediated by VEGFA/Snail1 axis in ARPE-19 cells, which may indirectly up-regulate YY1 by targeting Smurf2. Compared with healthy subjects, patients with uncomplicated T2D, NPDR, and PDR had significantly increased blood NR2F1-AS1. Furthermore, NR2F1-AS1 can distinguish T2D individuals from healthy subjects and PDR individuals from NPDR individuals.^[35]^ NR2F1-AS1 also has a strong ability to predict PDR and may be involved in the progression of DR by regulating EndMT.^[36]^ MEF2A can mediate the migration, tube formation, and apoptosis of retinal vascular endothelial cells through cZNF609, thus participating in the process of retinal vascular injury and neovascularization.^[37]^ NEAT1 regulates the development of DR epithelial-mesenchymal transformation through sex-determining region Y correlation high mobility group box 4 (SOX4), and the miR-204/SOX4 signal pathway is an important pathway associated with DR.^[38]^ The regulatory mechanism of these TFs requires further investigation.

CSF-1 and its receptor CSF1R are mainly expressed in microglia.^[39]^ Microglia play an important role in the immune regulation process in DR lesions. The signal transduction pathway of CSF-1/CSF1R is an important regulatory pathway that mediates the chemotaxis, proliferation, and M2 type polarization of macrophages. It may be a key pathway in the proliferation, migration, and angiogenesis of vascular endothelial cells. In many tumors, CSF1 and its receptor CSF-1R are highly expressed, and inhibition of migration and infiltration of tumor tissue can be observed after inhibiting CSF1.^[40]^

Mitogen activated protein kinase signal (MAPK) is one of the most important signaling systems in the body. It is a serine/threonine protein kinase, and the ERK signaling pathway is a key mitogen kinase in cell signaling, transmitting mitogen signals.^[41]^ The ERK signaling pathway follows 3 stages of the MAPK cascade reaction: the Ras-Raf MEK ERK pathway. Ras-Raf-MEK-ERK pathway is one of the important signal transduction pathways involved in physiological processes such as cell growth, development, division, migration, metabolism, and apoptosis.^[41]^ The phosphorylated ERK can convert extracellular stimulation signals into intracellular reactions, promote phosphorylation of multiple TFs in the nucleus, and enhance transcription activity. ERK is involved in tumor genesis by mediating the degradation of extracellular matrix, adhesion and movement of tumor cells, and tumor angiogenesis.^[41]^ Various growth factors, ions, and hydrogen peroxide can activate the ERK pathway through phosphorylation. In vitro experiments have demonstrated that ERK signaling pathway is involved in endothelial cell proliferation and angiogenesis.

Because ERK is activated to regulate p-ERK1/2 in the signal pathway, this study selected p-ERK1/2 as the research object. In this study, we observed that in high glucose environments, the mRNA and protein expressions of CSF1R and p-ERK1/2 in RF/6A increased, with a trend of positive correlation. It is consistent with the results of the first part of bioinformatics analysis and the second part of CSF1R expression in the vitreous of DR patients. It is further confirmed that the CSF1R/p-ERK1/2 signaling pathway plays a catalytic role in the occurrence and development of DR, and CSF1R can be used as a new target for DR diagnosis and treatment research.

This study only verified the expression of CSF1R and p-ERK1/2 under high glucose environment in RF/6A. When the expression of CSF1R changes, the expression of p-ERK1/2 and the apoptosis of cells may change. Further research should be conducted on the expression of p-ERK1/2 and the apoptosis of cells when CSF1R is inhibited or overexpressed. This study is only to verify the results in vitro experiments. Later, we should increase in vivo experiments to build an animal model of diabetes. After sampling, we should further analyze and study the expression of CSF1R, p-ERK1/2 and M2 macrophages, and inhibit or overexpress CSF1R in the eyes of animal models. After the expression changes, we should observe the expression of p-ERK1/2 and M2 macrophages and the distribution of retinal blood vessels. Through in vitro and in vivo experiments, a more comprehensive study was conducted on the effects of CSF1R/p-ERK1/2 axis and M2 macrophages on the pathogenesis of DR.

## 5. Conclusion

This investigation to the best of our knowledge is the first to use bioinformatics tools to study immune-related mechanisms in DR pathological process and screened 168 DEIRGs, and 15 hub genes, all enriched in M2 macrophage-related pathways. This research verified that the expression trend of some of these genes is consistent with the predicted results of bioinformatics analysis. However, further research is needed to confirm the function of and the relationship between target genes and the immune infiltration spectrum in the development of DR. CSF1R and p-ERK1/2 are highly expressed in rhesus monkey chorioretinal endothelial cells in high glucose environments, and there is a positive correlation between CSF1R and p-ERK1/2 expression. It is speculated that there is a CSF1R/p-ERK1/2 signaling pathway in RF/6A in high glucose environments, and it plays a role in promoting the development of retinal neovascularization.

## Author contributions

**Data curation:** Linhui Yuan.

**Formal analysis:** Linhui Yuan.

**Investigation:** Linhui Yuan.

**Methodology:** Linhui Yuan, Sheng Li.

**Resources:** Lijun Zhang, Sheng Li, Jixin Zou.

**Software:** Linhui Yuan.

**Supervision:** Xin Liu.

**Validation:** Xin Liu.

**Writing – original draft:** Linhui Yuan.

**Writing – review & editing:** Linhui Yuan, Lijun Zhang.
